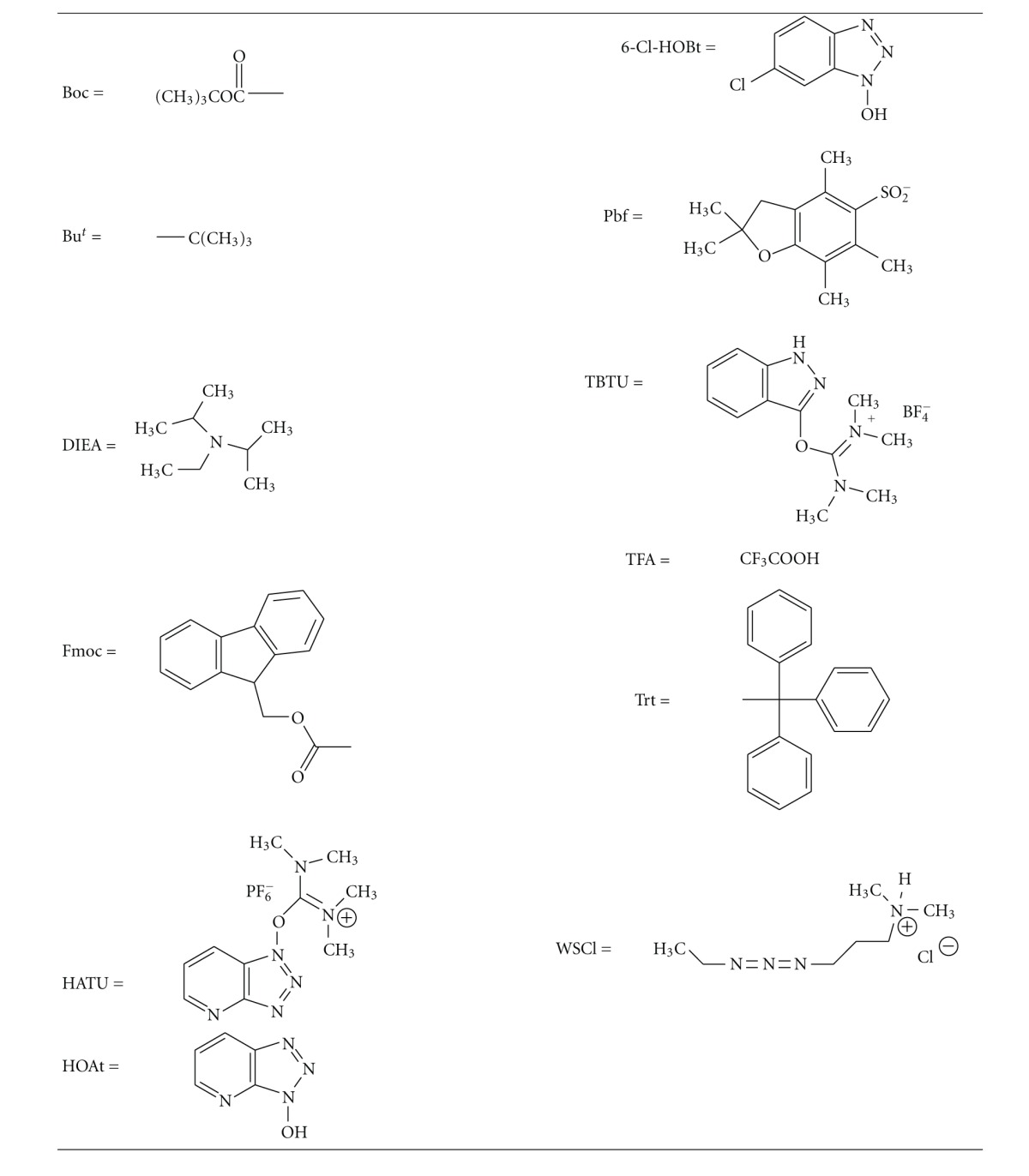# Synthesis of Hemopressin Peptides by Classical Solution Phase Fragment Condensation

**DOI:** 10.1155/2012/186034

**Published:** 2012-11-27

**Authors:** P. Anantha Reddy, Sean T. Jones, Anita H. Lewin, F. Ivy Carroll

**Affiliations:** Center for Organic and Medicinal Chemistry, Discovery Sciences Research Triangle Institute, Research Triangle Park, NC 27709-2194, USA

## Abstract

A fragment condensation solution phase assembly of the naturally occurring CB_1_ inverse agonist nonapeptides, Pro-Val-Asn-Phe-Lys-Phe/Leu-Leu-Ser-His-OH (hemopressins), and two other homologues: N-terminal 2-amino acid (dipeptide) extended undecapeptide, Val-Asp-Pro-Val-Asn-Phe-Lys-Leu-Leu-Ser-His-OH, and three-amino acid (tripeptide) extended dodecapeptide, Arg-Val-Asp-Pro-Val-Asn-Phe-Lys-Leu-Leu-Ser-His-OH, both CB_1_ agonists, is reported.

## 1. Introduction

**Figure 1:**
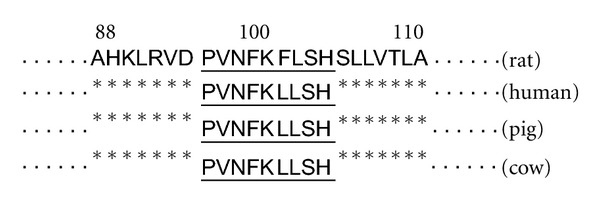
Sequence alignments of the *α*_1_-globin chain from various species. The sequence of hemopressin is underlined. Asterisks (*) indicate identical amino acids.

 Naturally occurring nonapeptides, Pro-Val-Asn-Phe-Lys-Phe/Leu-Leu-Ser-His-OH (hemopressins), derived from the *α*_1_ chain of hemoglobin of rat, human, pig, and cow are inverse agonists at the cannabinoid CB_1_ receptor [1]. Sequence alignments of hemopressins from various species differ only at position 100 of the *α*_1_-globin chain (Figure 1) where Phe (**F**) in rat is replaced by Leu (**L**) in human, pig, and cow sequences [2].

For convenience, hereafter, nonapeptide Pro-Val-Asn-Phe-Lys-Phe-Leu-Ser-His-OH, isolated from rat hemoglobin, is abbreviated as rHP and Pro-Val-Asn-Phe-Lys-Leu-Leu-Ser-His-OH, isolated from human, pig, and cow, as hHP. Interestingly, the N-terminally extended homologues of hHP: Val-Asp-Pro-Val-Asn-Phe-Lys-Leu-Leu-Ser-His-OH (VD-hHP) and Arg-Val-Asp-Pro-Val-Asn-Phe-Lys-Leu-Leu-Ser-His-OH (RVD-hHP), are in fact found to be CB_1_ agonists [3, 4]. In addition, hemopressin was recently shown to self-assemble into fibrils [5] at physiological pH. Since peptide amyloid fibril formation is implicated in Alzheimer's and Parkinson's diseases, these relatively small peptides deserve a systematic investigation into their structure activity relationships (SARs). 

 Towards this objective, and to be able to produce the desired truncated peptides for SAR studies, a solution phase fragment condensation was adopted to synthesize these peptides and other homologues.

**Scheme 1:**
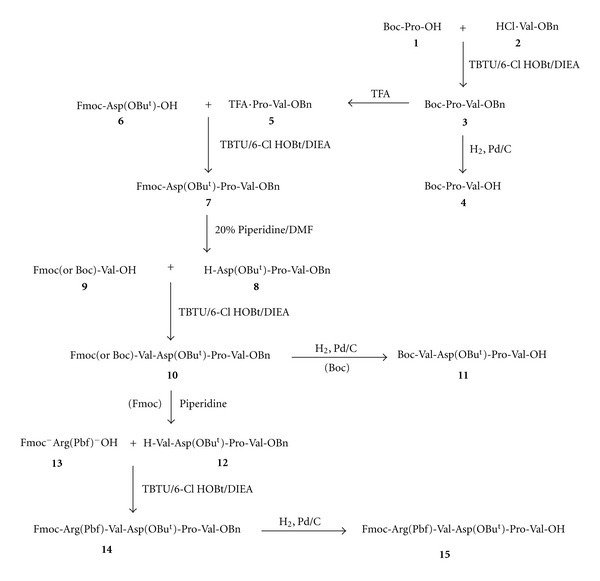
Synthesis of hemopressin (part 1).

**Scheme 2:**
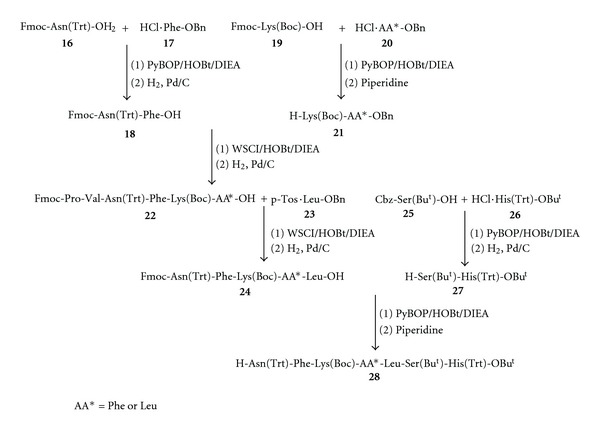
Synthesis of hemopressin (part 2).

 Though the solid phase synthesis is a fast route to synthetic peptides, the classical solution phase approach by fragment condensation has many advantages where several truncated peptides are available with minimum effort for structure activity investigations. In addition, peptide fragments from solution phase synthesis can be purified, and the pure intermediates are elaborated to the desired target peptide. Also, the smaller fragments used to make larger peptides give way for easier purifications (by size exclusion chromatography) of the target peptides by taking advantage of their size differences. Here, we report the synthetic routes to hemopressin and other related peptides using this approach. The protecting groups (Table 1) used here are orthogonal. Both Boc and Fmoc chemistries were used where necessary in the synthesis of peptide fragments. Thus, the key peptide intermediates: Boc-Pro-Val-OH (**4**), Boc-Val-Asp(OBu^t^)-Pro-Val-OH (**11**), Fmoc-Arg(Pbf)-Val-Asp(OBu^t^)-)-Pro-Val-OH (**15**) (Scheme 1), the N-terminal fragments and H-Asn(Trt)-Phe-Lys(Boc)-Phe/Leu-Leu-Ser(Bu^t^)-)His(Trt)-OBu^t^)-(**28**) (Scheme 1 and Scheme 2), and the C-terminal fragment common to all three peptides were prepared [6, 7] as outlined. [For the synthesis of shorter peptides, syntheses were conducted on 15 mmol to 20 mmol scale; fragment condensations were performed on 2 mmol to 5 mmol scale. The coupling agents TBTU and PyBOP were used in the preparation of shorter peptide fragments, while HATU/HOAt/DIEA (DMF) was used in the condensations of the protected peptide fragments to target peptides. In all cases, the reaction times are 3 h to 4 h. Deprotection was carried out by exposure (40 min to 1 h) to 50% TFA/CH_2_Cl_2_ for Boc/Trt groups, 20% piperidine/DMF for Fmoc group and neat TFA, to deblock the Bu^t^)-ether/ester protecting groups. Product yields in the preparation of shorter fragments were moderately high (80% to 95%), while yields in the condensation of fragments to get the larger peptides were in the range of 50% to 70%.]

The dipeptide fragment Boc-Pro-Val-OH (**4**) was prepared in two steps by condensation of **1** with **2** followed by hydrogenolysis of the resulting intermediate **3** (Scheme 1). Preparation of key intermediate **11** involved five easy steps (Scheme 1). Briefly, intermediate **5,** obtained by treatment of intermediate **3** with TFA, was condensed with **6** to give intermediate **7**, which after removal of the Fmoc group was coupled with **9** to provide intermediate **10**. Conversion of intermediate **10** to the desired key tetrapeptide intermediate, Boc-Val-Asp(OBu^t^)-Pro-Val-OH (**11**), was accomplished by hydrogenolysis. For the synthesis of the third key N-terminal pentapeptide, Fmoc-Arg(Pbf)-Val-Asp(OBu^t^)-Pro-Val-OH (**15**), intermediate **10** was converted to **12** by exposure to piperidine followed by coupling with Fmoc-Arg(Pbf)-OH (**13**) to give **14, **which on hydrogenolysis afforded **15**.

Next, the final C-terminal heptapeptide fragment, H-Asn(Trt)-Phe-Lys(Boc)-Phe/Leu-Leu-Ser(Bu^t^)-)-His(Trt)-OBu^t^)-(**28**), which is common to all target peptides was assembled as outlined in Scheme 2. The dipeptides, Fmoc-Asn(Trt)-Phe-OH (**18**) and H-Lys(Boc)-AA-OBn (**21**), where AA is either Phe or Leu, were prepared from condensations of **16** with **17** and **19** with **20**, respectively. Further, condensation of dipeptide **18** with dipeptide **21** provided tetrapeptide **22 **after hydrogenolysis. Elaboration of **22** to pentapeptide **24** was accomplished by addition of C-terminal residue **23** followed by hydrogenolysis (Scheme 2). The dipeptide fragment at the C-terminus, H-Ser(Bu^t^)-)-His(Trt)-OBu^t^)-(**27**), was assembled by coupling Cbz-Ser(Bu^t^)-)-OH (**25**) with HCl·His(Trt)-OBu^t^)-(**26**) followed by removal of the Cbz group. The final steps in the assembly of C-terminal heptapeptide fragment **28 **involve the (5 + 2) fragment condensation of pentapeptide **24** with dipeptide **27** followed by exposure to piperidine.

**Scheme 3:**
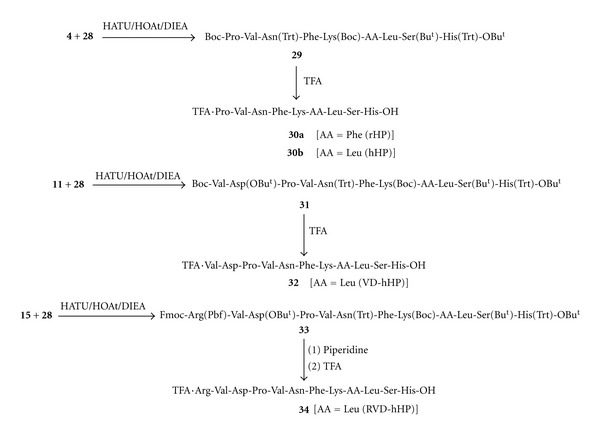
Synthesis of hemopressin (part 3).

 As shown in Scheme 3, using a (2 + 7) fragment condensation, dipeptide fragment **4** was condensed with heptapeptide fragment **28** under HATU/HOAt/DIEA coupling conditions [8] to give the fully protected nonapeptide Boc-Pro-Val-Asn(Trt)-Phe-Lys(Boc)-Phe/Leu-Leu-Ser(Bu^t^)-His(Trt)-OBu^t^ (**29**). The later individual nonapeptide(s) (**29**) on exposure to TFA furnished the target peptide(s) Pro-Val-Asn-Phe-Lys-Phe/Leu-Leu-Ser-His-OH (hemopressin) (**30a/30b**).

 Similarly, VD-hemopressin (**32**) and RVD-hemopressin (**34**) were also assembled using (4 + 7) and (5 + 7) fragment condensations, respectively (Scheme 3) involving intermediates **11**, **15**, **28**, **31**, and **33**. [Representative fragment coupling procedure for the synthesis of VD-Hemopressin: to a solution of Boc-Val-Asp(OBu^t^)-Pro-Val-OH (**11**) (1.4 g, 2 mmol), 6-Cl-HOBt (0,34 g, 2 mmol) in CH_2_Cl_2_ (60 mL) was added as TBTU reagent (0.65 g, 2 mmol) in CH_2_Cl_2_ (25 mL). To this mixture was added Asn(Trt)-Phe-Lys(Boc)-Leu-Leu-Ser(Bu^t^)-)-His(Trt)-OBu^t^)-(**28**) (3.2 g, ~2 mmol) in CH_2_Cl_2_ (50 mL). The mixture was stirred overnight at room temperature. After an acid base workup, the fully protected peptide, Boc-Val-Asp(OBu^t^)-Pro-Val-Asn(Trt)-Phe-Lys(Boc)-Leu-Leu-Ser(Bu^t^)-)-His(Trt)-OBu^t^)-(**31**) (2.6 g), was isolated. The crude peptide was purified by gel filtration on Sephadex LH-20 using MeOH. The purified product was exposed to 50% TFA/CH_2_Cl_2_ to remove all the protecting groups to give VD-hemopressin, Val-Asp-Pro-Val-Asn-Phe-Lys-Leu-Leu-Ser-His-OH (**32**) (VD-hHP) (0.31 g) [MS (ESI) 1269.1 (M+H)]. The crude peptide was purified to homogeneity by preparative reversed phase HPLC. The HPLC conditions were as follows: Vydac C_18_ column (218TP1022); flow rate: 15 mL/min; using a gradient (10% B **→** 65% B over 30 min) where A = 0.1% TFA/H_2_O and B = 0.1% TFA/CH_3_CN; UV detection 220 nm.] All target peptides: TFA·Pro-Val-Asn-Phe-Lys-Phe-Leu-Ser-His-OH (rHP) (**30a**), TFA·Pro-Val-Asn-Phe-Lys-Phe-Leu-Ser-His-OH (hHP) (**30b**), TFA·Val-Asp-Pro-Val-Asn-Phe-Lys-Phe-Leu-Ser-His-OH (VD-hHP) (**32)**, and TFA·Arg-Val-Asp-Pro-Val-Asn-Phe-Lys-Phe-Leu-Ser-His-OH (RVD-hHP) (**34**), were purified to homogeneity by preparative reversed phase HPLC and characterized by TLC, HPLC, MS (ESI), and amino acid analysis. [(a) **rHP**: MS (ESI) m/z 1089.3 (M+H); [*α*]_D_^22^ −26° (c 0.2, MeOH); amino acid analysis: found *(calculated*) Pro, 1.10 *(1.00*); Val 0.96 *(1.00*); Asn, 0.72 *(1.00*); Phe, 2.00 *(2.00*), Lys, 0.61 *(1.00*), Leu, 1.30 *(1.00*), Ser, 1.13 *(1.00*), His, 0.26 *(1.00*); (b) **hHP**: MS (ESI) m/z 1055 (M+H); [*α*]_D_^22^ −5.3° (c 0.15, MeOH); amino acid analysis: found (*calculated*) Pro, 1.03 (*1.00*); Val 0.96 (*1.00*); Asn, 1.00 (*1.00*); Phe, 0.84 (*1.00*), Lys, 1.03 (*1.00*), Leu, 2.03 (*2.00*), Ser, 0.94 (*1.00*), His, 0.97 (*1.00*); (c) **VD**-**hHP**: MS (ESI) m/z 1269.1 (M+H), m/z 635.5 (M+H)^+2^; [*α*]_D_^22^ −21° (c 0.1, MeOH); amino acid analysis: found (*calculated*) Pro, 1.00 (*1.00*); Val 1.90 (*2.00*); Asx, 2.13 (*2.00*); Phe, 1.00 (*1.00*), Lys, 1.06 (*1.00*), Leu, 1.96 (*2.00*), Ser, 0.89 (*1.00*), His, 0.91 (*1.00*); (d) **RVD**-**hHP**: MS (ESI) m/z 1424.81 (M+H), m/z 712.90 (M+H)^+2^, m/z 475.60 (M+H)^+3^; [*α*]_D_^22^−35° (c 0.520, MeOH); amino acid analysis: Found (*calculated*) Pro, 0.90 (*1.00*); Val 1.90 (*2.00*); Asx, 2.2 (*2.00*); Arg, 0.80 (*1.00*); Phe, 1.10 (*1.00)*, Lys, 1.20 (*1.00*), Leu, 1.90 (*2.00*), Ser, 0.80 (*1.00*), His, 1.00 (*1.00*).] 

## Figures and Tables

**Table 1:**